# Ni_0.6_Zn_0.4_O Synthesised via a Solid-State Method for Promoting Hydrogen Sorption from MgH_2_

**DOI:** 10.3390/ma16062176

**Published:** 2023-03-08

**Authors:** Noratiqah Sazelee, Muhamad Faiz Md Din, Mohammad Ismail

**Affiliations:** 1 Energy Storage Research Group, Faculty of Ocean Engineering Technology and Informatics, University Malaysia Terengganu, Kuala Nerus 21030, Malaysia; atiqahsazelee19@gmail.com; 2 Department of Electrical and Electronic Engineering, Faculty of Engineering, National Defence University of Malaysia, Kem Sungai Besi, Kuala Lumpur 57000, Malaysia; faizmd@upnm.edu.my

**Keywords:** metal oxide, nickel zinc oxide, magnesium hydride, solid-state hydrogen storage

## Abstract

Magnesium hydrides (MgH_2_) have drawn a lot of interest as a promising hydrogen storage material option due to their good reversibility and high hydrogen storage capacity (7.60 wt.%). However, the high hydrogen desorption temperature (more than 400 °C) and slow sorption kinetics of MgH_2_ are the main obstacles to its practical use. In this research, nickel zinc oxide (Ni_0.6_Zn_0.4_O) was synthesized via the solid-state method and doped into MgH_2_ to overcome the drawbacks of MgH_2_. The onset desorption temperature of the MgH_2_–10 wt.% Ni_0.6_Zn_0.4_O sample was reduced to 285 °C, 133 °C, and 56 °C lower than that of pure MgH_2_ and milled MgH_2_, respectively. Furthermore, at 250 °C, the MgH_2_–10 wt.% Ni_0.6_Zn_0.4_O sample could absorb 6.50 wt.% of H_2_ and desorbed 2.20 wt.% of H_2_ at 300 °C within 1 h. With the addition of 10 wt.% of Ni_0.6_Zn_0.4_O, the activation energy of MgH_2_ dropped from 133 kJ/mol to 97 kJ/mol. The morphology of the samples also demonstrated that the particle size is smaller compared with undoped samples. It is believed that in situ forms of NiO, ZnO, and MgO had good catalytic effects on MgH_2_, significantly reducing the activation energy and onset desorption temperature while improving the sorption kinetics of MgH_2_.

## 1. Introduction

Due to its enormous energy density (142 MJ/kg), abundance, and completely clean combustion, hydrogen is gaining more attention as an alternative energy carrier [1,2]. However, the limited availability of effective storage solutions has prevented the widespread use of hydrogen. The three conventional systems for storing hydrogen are cryogenic liquid storage (5–10 bar, 253 °C), compressed gas storage (350–700 bar at ambient temperature), and solid-state storage [3,4]. For solid-state storage, hydrogen can be stored in a chemical hydride such as ammonia borane [5,6] or in metal hydrides such as MgH_2_, LiAlH_4_, NaAlH_4_, and other materials and is expected to have a high hydrogen capacity [7,8,9]. Nevertheless, MgH_2_ is appealing because of its abundance of resources, cheapness, and high gravimetric capacity (7.60 wt.%) [10,11,12]. The practical applications for MgH_2_ were still lacking because of sluggish kinetics, a high temperature (more than 400 °C), and a high dissociation enthalpy (ΔH = −74.5 kJ/mol) [13,14]. Several attempts have been conducted to overcome the drawbacks of MgH_2_, such as using the ball milling technique (to create smaller particles size) and doping with additives/catalysts including transition metals (Cu, Nb, Ti, Zn, Ni, and Co) and their compounds (likes carbides, fluorides, oxides and hydrides), nonmetallic materials (such as carbon nanotubes, graphene, carbon, and graphite), and intermetallics [15,16,17,18].

Ni is one of the effective catalysts for the MgH_2_ system. A previous study revealed that by synthesizing the Ni@rGO catalyst, the desorption temperature of MgH_2_ + 10 wt.% Ni_4_@rGO_6_ samples decreased by 61 °C [19]. Besides that, the MgH_2_ + 10 wt.% Ni_4_@rGO_6_ samples are capable of absorbing 5.00 wt.% H_2_ in 20 min at 100 °C and desorbing 6.10 wt.% of H_2_ at 300 °C within 15 min. A study led by Hou et al. [20] examined the role of NiMoO_4_ as a catalyst through the milling process in enhancing the performance of hydrogen storage MgH_2_. It is noteworthy that hydrothermal and sintering processes were used to produce NiMoO_4_. According to the findings, the in situ formation of Mg_2_Ni/Mg_2_NiH_4_ by NiMoO_4_ and MgH_2_ promotes the fast motion of hydrogen and boosts the hydrogen sorption performance of MgH_2_. Meng and co-workers [21] synthesized Ni@C by electrospinning technique and found that the MgH_2_–Ni@C samples released approximately 5.79 wt.% of H_2_ at 280 °C and 6.12 wt.% of H_2_ at 300 °C, whereas milled MgH_2_ hardly decomposes under the same time frame. A previous study suggested a new method to analyze the impact of Ni nanopowder on the hydrogen storage performance of MgH_2,_ and it was found that within 10 min, MgH_2_–2 mol% Ni could absorb 5.30 wt.% of H_2_ at 300 °C [22].

Besides that, the formation of the reversible transition for Mg_2_Ni/Mg_2_NiH_4_ is another significant point to be made based on the Mg–Ni system which revealed a positive catalytic effect of MgH_2_ [23]. Another study stated that the in situ formation of Mg_2_NiH_4_ serves as a hydrogen pump to propel the absorption/desorption kinetics of MgH_2_, hence boosting the hydrogen storage performance of MgH_2_ [24]. This statement was also confirmed by Yang et al. [25]. According to Ying et al. [26], the Mg_2_Ni phase served as a catalyst for the hydrogen molecule dissociation, causing faster nucleation of MgH_2_. As reported by the deep-going Fu’s group [27], the active species of Mg_2_Ni/Mg_2_NiH_4_ during the heating process managed to improve the hydrogen sorption kinetics of MgH_2_ via the addition of FeNi_2_S_4_. At 310 °C, the absorption kinetics of MgH_2_ speed up after the addition of Ni and ZrO_2_ [28]. The samples can absorb 6.10 wt.% of H_2_, while sluggish kinetics can be observed for milled MgH_2_ (4.60 wt.%). Furthermore, by the method of reducing self-assembled layered double hydroxide and graphene oxide to create NiCu/rGO, Lie et al. [29] demonstrated good hydrogen sorption kinetics compared with milled MgH_2_. Mao and co-workers [30] exposed that MgH_2_ with NiCl_2_ shows better sorption properties than CoCl_2_-doped samples. They found out that MgH_2_/NiCl_2_ decomposes at 300 °C while MgH_2_/CoCl_2_ decomposes at 304 °C. Furthermore, MgH_2_/NiCl_2_ composite released 4.58 wt.% of H_2_ at 300 °C within 1 h, compared with 2.21 wt.% for MgH_2_/CoCl_2_ and 0.77 wt.% of H_2_ for pure MgH_2_.

Studies have pointed out another catalyst, which is Zinc (Zn) to accelerate the absorption/desorption kinetics of MgH_2_. The onset desorption temperature of MgH_2_ doped 3 mol% of ZnFe_2_O_4_ initiated at about 300 °C [31]. In addition, Polanski and Bystrzycki [32] observed that the addition of ZnO significantly accelerated the absorption kinetics at 325 °C in just 10 min and reduced the activation energy to 147 kJ/mol when compared with MgH_2_. Thus, it is highly fascinating to explore the combination of these transition metals (Ni and Zn), given their good impact on boosting the absorption/desorption kinetics of hydrides. Furthermore, it is founded that the combination of several metals can speed up the hydrogen storage properties of MgH_2_. Other researchers have exposed that adding 5 wt.% of Zr_70_Ni_20_Pd_10_ powders to MgH_2_ enhances the hydrogenation/dehydrogenation behaviors [33]. Accordingly, combining MgH_2_ with additives/catalysts such as CeNi_5_, NdNi_5_, YNi_5_, PrNi_5_, and SmNi_5_ showed faster absorption and desorption kinetics at 300 °C within 200 s and 1800 s, respectively [34]. El-Eskandarany et al. [35] came to the conclusion that adding the LaNi_3_ additive caused a decrease in the initial decomposition temperature to 579 K and the activation energy to 73.26 kJ/mol. In addition, Wu et al. [36] synthesized porous LaNiO_3_ using a precipitation-combustion method and found that 10 wt.% of LaNiO_3_ can absorb 5.10 wt.% of H_2_ within 60 s at 200 °C. The further study exposed that in situ formations of LaH_3_ and Mg_2_NiH_4_ during the heating process significantly enhanced the performance of hydrogen storage for MgH_2_.

Therefore, in this study, Ni_0.6_Zn_0.4_O was prepared by using the solid-state method. This additive was used in order to enhance the kinetics of absorption/desorption of MgH_2_. This research is expected to reveal the catalytic mechanism to give a better understanding of the reaction between Ni_0.6_Zn_0.4_O and MgH_2_. It is worth noting that Ni_0.6_Zn_0.4_O is first applied in MgH_2_ for solid-state hydrogen storage performance.

## 2. Materials and Methods

For the first part, Ni_0.6_Zn_0.4_O was synthesized by the solid-state method by using Ni (≥99% pure; Sigma Aldrich, St. Louis, MO, USA), citric acid (≥98% pure; Sigma Aldrich), and sinc oxide (<100 nm; Sigma Aldrich). All of these materials were ground together for 15 min using the following amounts: 0.1195 g of Ni, 0.1521 g of citric acid and 0.0326 g of zinc oxide. The sample was then calcined for 1 h at 1000 °C.

Next, Ni_0.6_Zn_0.4_O was used as an additive in order to improve the hydrogen storage performance of MgH_2_. For this step, all the handling processes, including weighing, were completed in a glove box (MBRAUN UNIlab) with a pure argon atmosphere to prevent oxidation. The different weight percentages of Ni_0.6_Zn_0.4_O samples were milled together by using a planetary ball mill (NQM-0.4) to produce MgH_2_-X wt.% Ni_0.6_Zn_0.4_O samples (where X = 5, 10, 15, and 20). In this experiment, commercial MgH_2_ was acquired from Sigma Aldrich (≥95% pure). The sample was milled at 400 rpm for 1 h (15 min of milling time, 2 min of resting time, and 3 cycles) at room temperature. Each milling consists of four balls made of steel, and the ball-to-powder weight ratio is equal to 40:1.

To analyze the onset desorption temperature and absorption/desorption kinetics of the samples, Sievert-type pressure composition temperature (Advanced Materials Corporation, Pittsburgh, PA, USA) was used. The samples were heated up to 450 °C from room temperature. Meanwhile, 33.0 atm and 1.0 atm of pressure were used for the absorption/desorption kinetics process, which was carried out at 250 °C and 300 °C, respectively. The differential scanning calorimetry (DSC) was examined using a Mettler Toledo (Columbus, OH, USA) TG/DSC 1 in an Argon gas flow at 50 mL/min with various heating rates (15, 20, 25 and 30 °C/min) applied. About 3–5 mg of samples were loaded into an alumina crucible and heated from room temperature to 450 °C.

Structural characterization was performed with the help of the X-ray diffraction (XRD; Rigaku Miniflex, Tokyo, Japan) technique with a Cu-kα radiation range of 20–80° and a speed of 2.00°/min. The morphology of the composite was characterized by using scanning electron microscopy (SEM; JEOL JSM-6360LA) and energy dispersive X-ray spectroscopy (EDS; JEOL JSM-6360LA). The bonding of the samples was investigated using Fourier transform infrared spectroscopy (IR Shimadzu Tracer-100, Kyoto, Japan). Each FTIR data were obtained by averaging 40 scans from 400 to 2700 cm^−1^, and Renishaw Raman spectroscopy was conducted at room temperature with a 0.1% power laser measurement. Pure MgH_2_ and milled MgH_2_ were also characterized by using all the instruments to compare the results between the samples.

## 3. Results and Discussion

**Figure 1:**
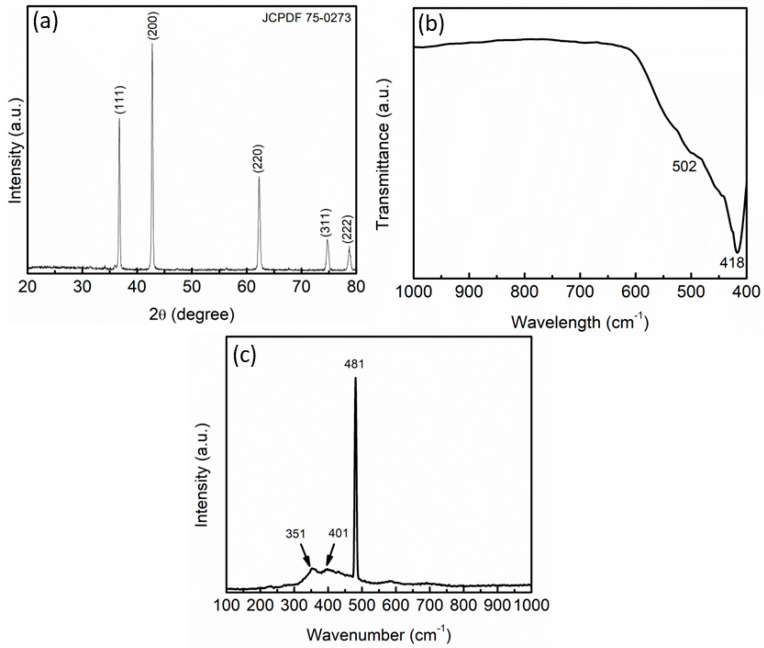
(**a**) XRD pattern, (**b**) FTIR spectra and (**c**) Raman spectra of Ni_0.6_Zn_0.4_O.

The XRD spectra of Ni_0.6_Zn_0.4_O samples prepared by the solid-state method are presented in Figure 1a. Referring to the reported standard Ni_0.6_Zn_0.4_O sample (JCPDF 75-0273), all reported diffraction peaks match it perfectly. Zinc oxide and nickel oxide, two potential impurity phases, were not found in the XRD spectra. The diffraction peaks at 36.85°, 42.81°, 62.15°, 74.49°, and 78.42° are used to represent the Ni_0.6_Zn_0.4_O crystal planes (111), (200), (220), (311), and (222), respectively. Similar observations were reported by Wei and co-workers [37]. The crystallite sizes (L) are estimated at 11.24 nm through the Scherrer formula as in Equation (1) below:

L = Kλ/*β* cos θ (1)

where λ is the X-ray used (0.154 nm), *β* (physical broadening) is the full width at half the maximum, θ is the angle of Bragg’s diffraction, and shape factor K = 0.94 constant. Figure 1b depicts the FTIR spectra of Ni_0.6_Zn_0.4_O, and the peaks at 418 cm^−1^ correspond to the Ni–O bond as suggested in the previous study [38,39]. Meanwhile, the peaks at 502 cm^−1^ are attributed to the Zn–O peaks as indicated by Raja et al. [40] and Handore et al. [41]. Furthermore, Raman spectra of Ni_0.6_Zn_0.4_O samples were present as in Figure 1c, clearly showing 3 distinct Raman bands. The peaks at 351 cm^−1^ and 401 cm^−1^ were attributed to the Zn–O peak, as proven by Bhunia et al. [42] and Marinho et al. [43]. Meanwhile, the peak at 481 cm^−1^ matches the Ni–O peak as exposed by Bose et al. [44].

**Figure 2:**
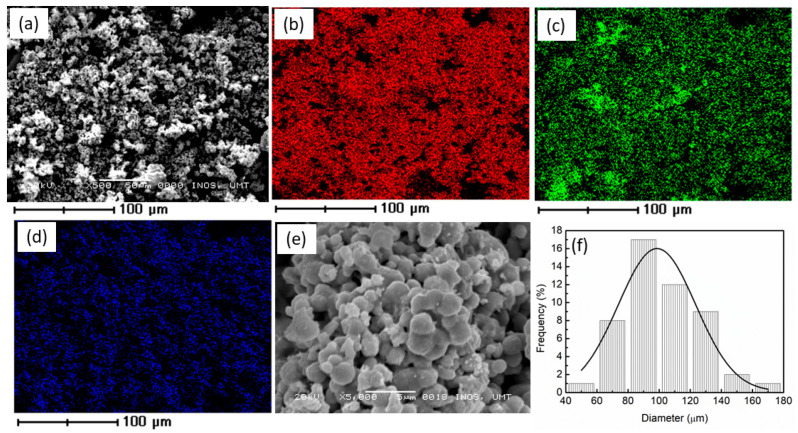
EDS images of the (**a**) Ni_0.6_Zn_0.4_O, (**b**) Ni, (**c**) Zn, (**d**) O, (**e**) SEM images and (**f**) Particle size distribution of Ni_0.6_Zn_0.4_O.

EDS characterization for Ni_0.6_Zn_0.4_O was conducted as shown in Figure 2 in order to recognize the existence and distribution of Ni_0.6_Zn_0.4_O. To evaluate the element distribution over a broad region, the magnification was set at 500×. The EDS mapping below shows the distribution of different elements. Figure 2a displays the Ni_0.6_Zn_0.4_O samples; Figure 2b,c show the Ni and Zn elements, respectively. However, Figure 2d illustrates the O element. From the result obtained, it is shown that elements Ni, Zn, and O are uniformly distributed. Table 1 below proved the element of the Ni_0.6_Zn_0.4_O. From the results of XRD, FTIR, Raman, and EDS mapping, it is proved that pure Ni_0.6_Zn_0.4_O was successfully synthesized by the solid-state method. The SEM images as presented in Figure 2e indicated that Ni_0.6_Zn_0.4_O has a spherical morphology and the particles were agglomerate. Meanwhile, Figure 2f shows the results of the particle size distribution (PSD) analysis. The PSD was analyzed using Image J. Most of the particles are concentrated at a size of approximately 98.6 µm.

**Figure 3:**
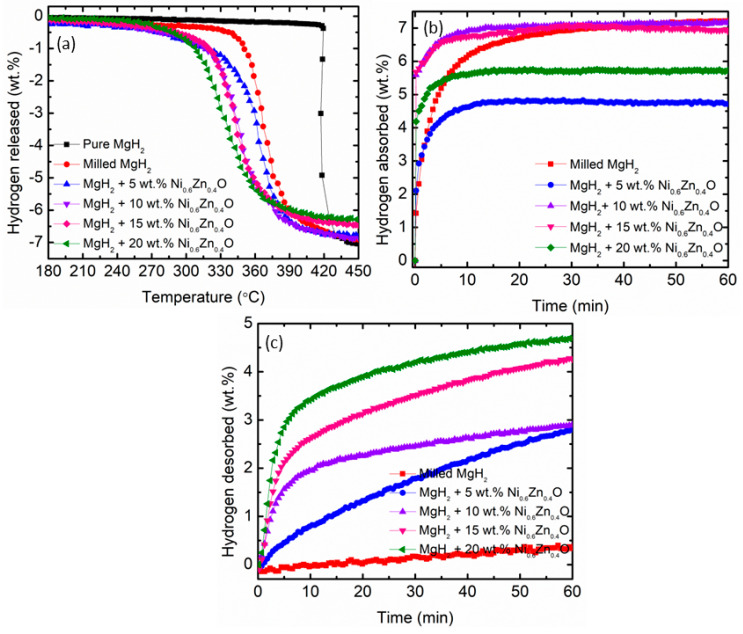
(**a**) Temperature-programmed-desorption curves, (**b**) Absorption kinetics at 250 °C, 33.0 atm and (**c**) Desorption kinetics at 300 °C, 1.0 atm.

Figure 3a exhibited the temperature-programmed desorption curves of pure MgH_2_, milled MgH_2_, and MgH_2_ doped with different weight percentages (5, 10, 15, and 20) of Ni_0.6_Zn_0.4_O. The pure MgH_2_ decomposes at 418 °C with an approximate 7.10 wt.% total dehydrogenation capacity. Milling MgH_2_ for 1 h lowered the onset desorption temperature to 341 °C, 77 °C lower than pure MgH_2_. Remarkably, the onset desorption temperature reduces after the Ni_0.6_Zn_0.4_O additive is added. MgH_2_–5 wt.% Ni_0.6_Zn_0.4_O samples decompose at 280 °C with a 6.80 wt.% total H_2_ release. After the addition of 10 wt.%, 15 wt.%, and 20 wt.% of Ni_0.6_Zn_0.4_O as an additive with MgH_2_, the initial desorption temperatures were lowered to 285 °C, 305 °C, and 293 °C, respectively. In addition, as the amount of Ni_0.6_Zn_0.4_O increased to 10 wt.%, 15 wt.% and 20 wt.%, the total hydrogen release declined to 6.80 wt.%. 6.50 wt.% and 6.30 wt.%, respectively. Numerous studies have shown that this trend was caused by the dead weight of the Ni_0.6_Zn_0.4_O. Research conducted by Bhatnagar et al. [45] stated that the total desorption capacity of MgH_2_–TiF_2_ is less than MgH_2_ because TiH_2_ somehow does not evolve H_2_ and acts as a dead weight for the MgH_2_–TiH_2_ system.

The absorption kinetics was conducted at 250 °C for 1 h, and the result also shows that the addition of the Ni_0.6_Zn_0.4_O additive enhances the performance of MgH_2,_ as proved in Figure 3b. The results showed that within only 5 min, milled MgH_2_ was able to absorb 4.80 wt.% of H_2_. The addition of 10 wt.%, and 15 wt.% of Ni_0.6_Zn_0.4_O with MgH_2_ increased their absorption capacity to 6.50 wt.% of H_2_ in the same amount of time. A slight increment in the absorption capacity for MgH_2_–20 wt.% Ni_0.6_Zn_0.4_O samples can be observed, which is 5.40 wt.%. However, MgH_2_–5 wt.% Ni_0.6_Zn_0.4_O samples showed the lowest amount of absorption capacity, which is 4.10 wt.% under the same circumstances. The absorption kinetics of MgH_2_ with another catalyst were also included for comparison purposes, as shown in Table 2.

Apart from the absorption behavior of undoped and doped samples, the hydrogen desorption kinetics of MgH_2_ doped with Xwt.% Ni_0.6_Zn_0.4_O (where X = 5, 10, 15, and 20) were conducted at 300 °C for 1 h, as shown in Figure 3c. It is apparent that MgH_2_ doped with Ni_0.6_Zn_0.4_O desorbed hydrogen significantly faster than that of milled MgH_2_. Milled MgH_2_ can desorb 0.02 wt.% of H_2_ while MgH_2_ doped with 5 wt.% of Ni_0.6_Zn_0.4_O can desorb 1.30 wt.% of H_2,_ and MgH_2_–10 wt.% Ni_0.6_Zn_0.4_O samples desorbed 2.30 wt.% of H_2_ within 20 min. As the Ni_0.6_Zn_0.4_O additive is increased to 15 wt.% and 20 wt.%, the total amount of hydrogen released rises to 3.10 wt.% and 3.90 wt.%, respectively.

The good catalytic effect of the Ni_0.6_Zn_0.4_O were clarified by the faster absorption/desorption kinetics of MgH_2_. According to Yang et al. [25], the Mg–H bond was significantly stretched by the Ni catalyst action, which is more favorable for H separation and can speed up the desorption rate of MgH_2_. It is clearly apparent that introducing Ni_0.6_Zn_0.4_O as an additive will significantly reduce the onset desorption temperature and enhance the absorption/desorption kinetics of MgH_2_ as summarized in Table 3 below. Pure MgH_2_ and milled MgH_2_ were also included for comparison. Considering the influence of the Ni_0.6_Zn_0.4_O as an additive on the onset desorption temperature and sorption kinetics, MgH_2_–10 wt.% Ni_0.6_Zn_0.4_O samples as an additive were selected for further study.

Using kinetic models to represent the behavior of absorption and desorption is a great idea to gain a better understanding of the kinetic mechanism in MgH_2_–10 wt.% Ni_0.6_Zn_0.4_O samples. In 2010, Luo et al. [50] investigated two kinds of kinetic models, which are the Jander model and the Chou model, on the hydriding kinetics of Mg-Ni based alloys. For instance, Cheng et al. [51] proposed a kinetic model based on the characteristics of desorption time for TiVNbCr alloy using the Jander diffusion model, the Ginstling-Brounshtein model, and the Johnson-Mehl-Avrami-Kolmogorov (JMA) equation. Furthermore, JMA plots of the Mg_90_Ce_5_Y_5_ alloy with various catalysts such as MoO_3_, MoO_2_, and Mo were explored by Wang and co-workers [52]. In this study, the kinetic models of JMA and Contracting Volume (CV) were analyzed as can be seen in Table 4 [53]. According to Pang and Li [54], these models were chosen because they accurately fit the experimental data and did not require any additional approximations or assumptions. Additionally, these models have been used by other researchers to comprehend the rate-limiting steps of the material.

**Figure 4:**
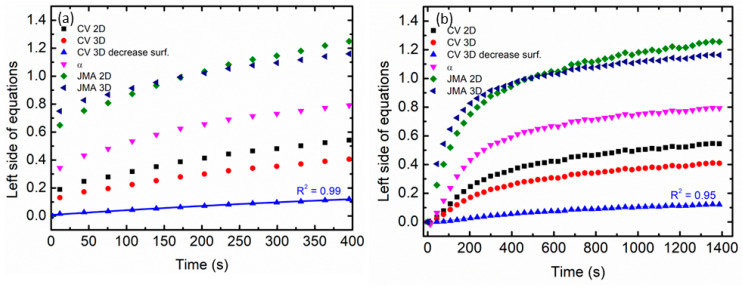
The calculation of the various kinetic equations for MgH_2_–10 wt.% Ni_0.6_Zn_0.4_O samples is shown in Table 4 for (**a**) absorption kinetics at 250 °C and (**b**) desorption kinetics at 300 °C.

In this context, the best linear plot of the absorption and desorption kinetics of MgH_2_–10 wt.% Ni_0.6_Zn_0.4_O samples with the kinetic equations in Table 4 determined the rate-limiting steps as shown in Figure 4a,b, respectively. The kinetic curves for the samples were measured for the reacted fraction in the range of 0 to 80%. As shown in the following figure, the CV 3D decrease surface can best explain the absorption and desorption kinetics at 250 °C and 300 °C, respectively.

**Figure 5:**
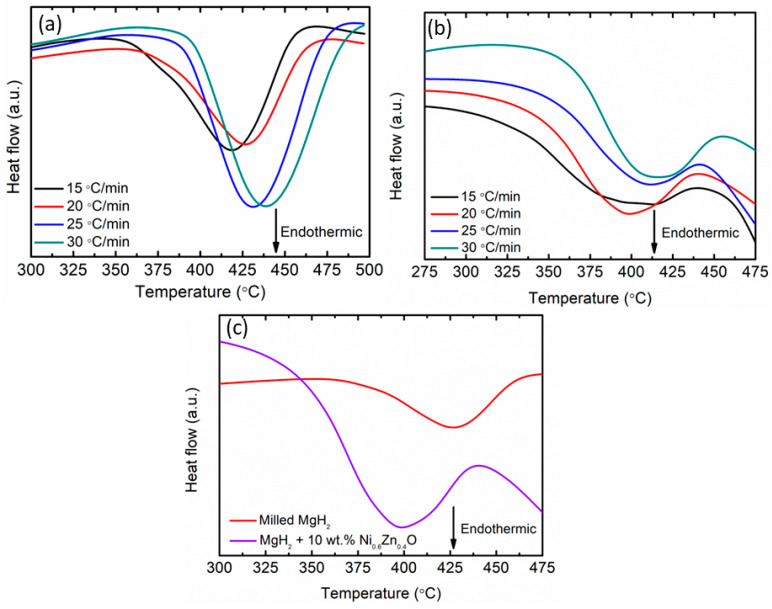
DSC traces for (**a**) milled MgH_2_, (**b**) MgH_2_–10 wt.% Ni_0.6_Zn_0.4_O samples at 15, 20, 25 and 30 °C/min and (**c**) DSC traces at 20 °C/min for milled MgH_2_ and MgH_2_–10 wt.% Ni_0.6_Zn_0.4_O samples.

The DSC curves for milled MgH_2_ and MgH_2_–10 wt.% Ni_0.6_Zn_0.4_O samples were evaluated at different heating rates, as represented in Figure 5a and Figure 5b, respectively. One endothermic peak is visible in both samples, indicating the decomposition of MgH_2_ to Mg. Increasing the heating rates resulted in an increase in the temperature of the samples. For comparison, DSC traces at 20 °C/min for milled MgH_2_ and MgH_2_–10 wt.% Ni_0.6_Zn_0.4_O samples were examined as in Figure 5c. From the result obtained, the temperature for milled MgH_2_ was 428 °C, while the MgH_2_–10 wt.% Ni_0.6_Zn_0.4_O samples were 397 °C. It is noticeable that the Ni_0.6_Zn_0.4_O additive affected the endothermic peak of hydrogen desorption to shift remarkably to a lower temperature. Besides, it was observed that the inclusion of Mg(Nb)O resulted in a reduction in the endothermic peak of MgH_2_, which is due to the weakening of Mg–H bonds caused by Mg(Nb)O [55].

**Figure 6:**
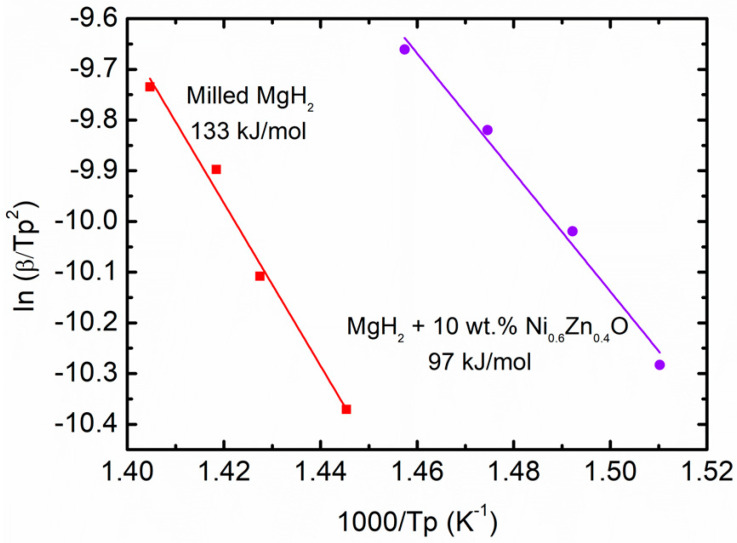
Activation energy for milled MgH_2_ and MgH_2_–10 wt.% Ni_0.6_Zn_0.4_O samples.

The remarkable effect of the Ni_0.6_Zn_0.4_O additive on the desorption kinetic properties of MgH_2_ was further examined by calculating the apparent activation energy (*E_A_*) using the Kissinger equation below (Equation (2)):

In [*β*/*T_p_*^2^] = −*E_A_*/R*T_p_* + A (2)

where *T_p_* is the peak temperature in the DSC curve, *β* is the heating rate of the samples, R is the gas constant, and A is a linear constant. Figure 6 revealed the Kissinger plots of the milled MgH_2_ and MgH_2_–10 wt.% Ni_0.6_Zn_0.4_O samples by fitting the data points. From the figure, the activation energy of milled MgH_2_ was 133 kJ/mol. However, in MgH_2_–10 wt.% Ni_0.6_Zn_0.4_O samples, the value was reduced to 97 kJ/mol. This number dropped by 36 kJ/mol. This revealed that the addition of Ni_0.6_Zn_0.4_O as an additive to MgH_2_ resulted in a notable decrease in the kinetic barrier desorption of the MgH_2_ system, which is beneficial for hydrogen release from MgH_2_. These findings are also consistent with earlier research that showed the addition of an additive or catalyst lowers the activation energy of MgH_2_ [56,57]. Zhang and co-workers [58] exposed that the reaction energy barrier for the desorption reduced to 109 kJ/mol when MnMoO_4_ was doped to MgH_2_. The apparent activation energy is roughly 30% lower than pure MgH_2_. According to research by Hu et al. [59], the addition of K_2_Ti_8_O_17_ can successfully lower the activation energy by 59 kJ/mol.

**Figure 7:**
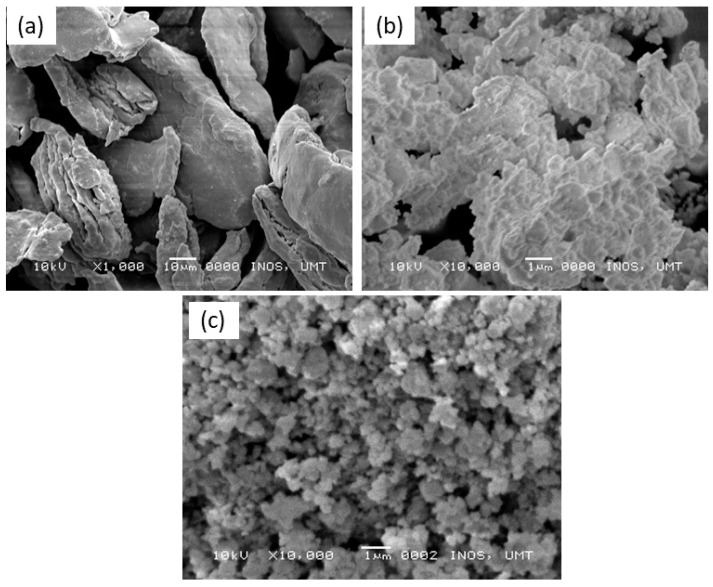
SEM images of (**a**) pure MgH_2_, (**b**) milled MgH_2_ and (**c**) MgH_2_–10 wt.% Ni_0.6_Zn_0.4_O samples.

Figure 7 below displays SEM images of the pure MgH_2_, milled MgH_2_, and MgH_2_–10 wt.% Ni_0.6_Zn_0.4_O samples. Pure MgH_2_ revealed the morphology of the sample as an irregular shape range larger than 50 µm as in Figure 7a. A similar outcome was discovered by Mahsa et al. [60]. They exposed that the morphology of pure MgH_2_ has irregular shapes with larger particles. It should be noted that smaller particle sizes can be observed after MgH_2_ is milled for 1 h, as presented in Figure 7b. This proved that the performance of MgH_2_ was also directly affected by the milling process. Next, changes in the morphological parameters of the powder can also be detected by Czujko et al. [61]. According to Shahi et al. [62], the onset desorption temperature of pure MgH_2_ decreased from 422 °C to 367 °C. It may be pointed out that the milling process of MgH_2_ for 25 h reduces the particle size of MgH_2_, thereby lowering the desorption temperature of MgH_2_. As expected, MgH_2_–10 wt.% Ni_0.6_Zn_0.4_O samples exhibited a smaller particle size as compared with milled MgH_2_ (as can be seen in Figure 7c). Ali et al. [56] introduced CoTiO_3_ to MgH_2_ and showed that the particle size of the composite changed to a finer and smaller size. According to the research results of Somo et al. [63], smaller particle sizes allow quick dissociation into the surface of materials. Besides that, the addition of Nb to MgH_2_ creates a large number of hydrogen diffusion channels and speeds up hydrogen flow along the MgH_2_/Mg interfaces, continuing to improve the sorption kinetics of MgH_2_ [64]. In light of this, it is obvious that adding Ni_0.6_Zn_0.4_O causes the particle size to be greatly decreased, which is useful for improving the performance of MgH_2_.

**Figure 8:**
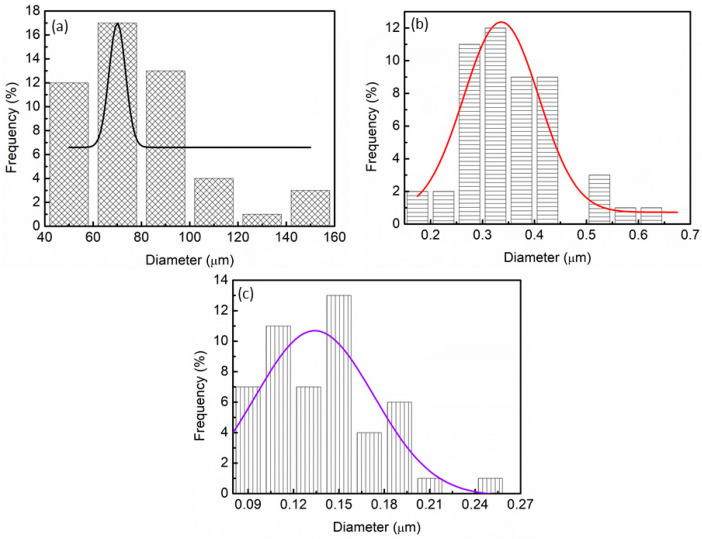
PSD of (**a**) pure MgH_2_, (**b**) milled MgH_2_ and (**c**) MgH_2_–10 wt.% Ni_0.6_Zn_0.4_O samples.

The PSD of pure MgH_2_, milled MgH_2_, and MgH_2_–10 wt.% Ni_0.6_Zn_0.4_O samples were analyzed using Image J (version 2022). As shown in Figure 8a, the PSD calculated for pure MgH_2_ was 84.8 µm. The calculated PSD for milled MgH_2_ decreased to 0.29 µm as shown in Figure 8b. A study led by Maddah et al. [65] exposed that the average particle size of MgH_2_ decreased from 30 µm to 2.2 µm. Furthermore, as the milling time is extended up to 30 h, no discernible difference is seen. However, in this study, the PSD was decreased to 0.13 µm when 10 wt.% of Ni_0.6_Zn_0.4_O was added to MgH_2_, as shown in Figure 8c. This demonstrated how significantly MgH_2_’s size was reduced after the addition of Ni_0.6_Zn_0.4_O as an additive. Moreover, Xiao and colleagues [66] stated that the particle size of milled MgH_2_ decreased to a range of 80 to 80 nm and lowered to 50 to 400 nm after LiCl was added.

**Figure 9:**
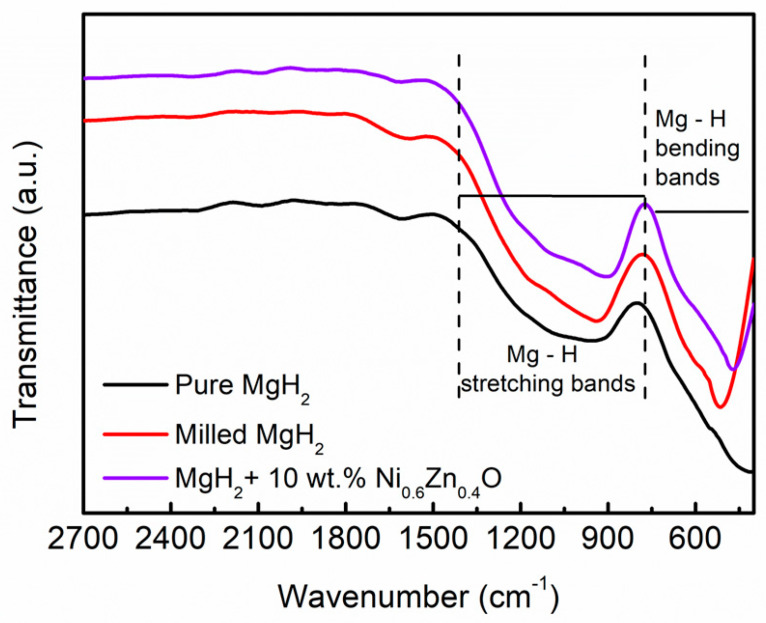
FTIR pattern of (**a**) pure MgH_2_, (**b**) milled MgH_2_ and (**c**) MgH_2_–10 wt.% Ni_0.6_Zn_0.4_O samples.

The effect of Ni_0.6_Zn_0.4_O addition on the MgH_2_ bonding was investigated by using FTIR, as shown in Figure 9. All the samples exhibited two bands: (i) 400–800 cm^−1^, corresponding to Mg–H bending bands, and (ii) 800–1400 cm^−1^, attributed to the Mg–H stretching bands as previously shown by Zhang et al. [67]. For milled MgH_2_, an obvious peak around 515 cm^−1^ is attributed to Mg–H bending bands. This peak indicated that the milled MgH_2_ was stable during the milling process. In our study, the bending and stretching bands were at about 772 cm^−1^ and 1380 cm^−1^, respectively. No new peak was detected due to the low amount of Ni_0.6_Zn_0.4_O as an additive. However, after the addition of 10 wt.% Ni_0.6_Zn_0.4_O as an additive, the peaks were shifted to a low wavenumber, which indicates the weakness of the Mg–H bond. Furthermore, Ismail et al. [68] also agreed with these findings.

**Figure 10:**
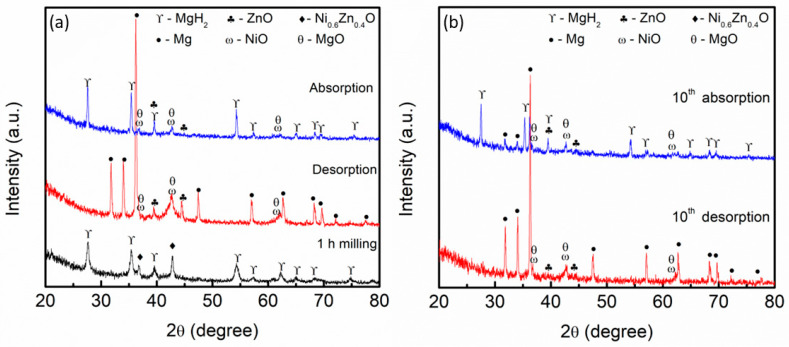
XRD pattern of MgH_2_–10 wt.% Ni_0.6_Zn_0.4_O samples (**a**) after 1st cycle and (**b**) after 10th cycle.

The XRD pattern of the MgH_2_–10 wt.% Ni_0.6_Zn_0.4_O samples after milling for 1 h, after desorption at 450 °C, and after absorption at 250 °C at the 1st cycle is exhibited in Figure 10a. As shown in Figure 10, the peaks of Ni_0.6_Zn_0.4_O and MgH_2_ were present, which indicates the parent materials of the composite. Meanwhile, after MgH_2_–10 wt.% Ni_0.6_Zn_0.4_O samples were heated at 450 °C, as exhibited in the figure below (labelled desorption), the peaks of MgH_2_ and Ni_0.6_Zn_0.4_O disappeared. Peak Mg was present, which revealed that MgH_2_ was fully decomposed to Mg as exhibited in the equation below:

MgH_2_ → Mg + H_2_ (3)

New peaks of ZnO, NiO, and MgO could also be seen as the samples were heated up. However, the peaks of Mg were completely transformed into MgH_2_ during the absorption process at 250 °C, while the peaks of ZnO, NiO, and MgO remained unaltered (labeled absorption).

The XRD pattern for MgH_2_–10 wt.% Ni_0.6_Zn_0.4_O samples after the 10th cycle of desorption and absorption was analyzed and illustrated as in Figure 10b. Obviously, the Mg peak dominates even at the 10th cycle, and no peak of MgH_2_ was found, as demonstrated in the figure below (labeled 10th desorption). However, the peaks of ZnO, NiO, and MgO remained unchanged even after the 10th cycle. Another peak of the XRD spectra for absorption at 10th cycles was also reported in Figure 10b below, labeled 10th absorption. The peaks of MgH_2_ were found, which revealed the Mg peaks were transformed into MgH_2_. Nevertheless, the in situ forms of ZnO, NiO, and MgO still appeared and remain unchanged. Based on the result obtained, the in situ formation may also provide a significant effect that will help boost the hydrogen sorption performance of MgH_2_.

A previous work discovered that the performance of hydrogen storage MgH_2_ is significantly improved by the inclusion of metal oxide as a catalyst or additive [69]. According to a study by Zou et al. [70], the polarization might weaken the Ti–O bonds and Mg-H bonds, which make MgH_2_ decompose quickly after the addition of TiO. Furthermore, Huang et al. [71] discovered that faster absorption/desorption kinetics of MgH_2_ can be observed after the addition of Sc_2_O_3_ and TiO_2_. Further findings indicate that the surface defects and grain boundaries created by the milling process after the addition of Sc_2_O_3_ and TiO_2_ provide a significant number of diffusion channels and active sites that greatly enhance the kinetics of MgH_2_.

In this study, the in situ formation of MgO, NiO, and ZnO was observed during the heating process of MgH_2_–10 wt.% Ni_0.6_Zn_0.4_O samples. The formation of MgO after the addition of additive/catalysts has well agreed with previous research. Aguey-Zinsou et al. [72] indicated that the role of MgO is rationalized in the concept of a “Process Control Agent”. On top of that, MgO has dispersed properties and good lubricant thus preventing MgH_2_ from clumping together. Additionally, Shan et al. [73] also revealed that one of the final reaction products of CoFe_2_O_4_ and MgH_2_ is MgO, which may help reduce the onset desorption temperature from 440 °C for as-received MgH_2_ to 160 °C after doping with 7 mol% of CoFe_2_O_4_. In order to tailor MgH_2_ performance, Ali et al. [74] introduced 10 wt.% of MgNiO_2_ to MgH_2,_ and the results show that MgH_2_–10 wt.% MgNiO_2_ samples can desorb roughly 5.10 wt.% of H_2_ within 10 min at 320 °C and begin to decompose at 258 °C. Surprisingly, at 200 °C, MgH_2_–10 wt.% MgNiO_2_ samples continue to absorb 6.10 wt.% of H_2_ in just 10 min. The performance of MgH_2_ as a hydrogen storage material is boosted by the formation of new MgO and NiO compounds.

A previous study reported that adding a Co_2_NiO catalyst can lower the desorption temperature by 117 °C (pure MgH_2_) and 70 °C (milled MgH_2_) and decrease the activation energy by 65 kJ/mol and 15 kJ/mol for pure MgH_2_ and milled MgH_2_, respectively [48]. According to a study by Zhang et al. [75], the bond between Mg and H is weaker than the bond between transition metals such as Ni. The release of the H atom and H_2_ recombination from the MgH_2_ surface is encouraged by the weakening of the bond between H and Mg caused by the strong bonding between Ni and H. Besides, Patah et al. [76] also exposed the fact that adding ZnO to MgH_2_ reduces the onset desorption peak of the DSC curves from 375 °C to 360 °C. Along this line, it is valuable to conclude that the addition of Ni_0.6_Zn_0.4_O as an additive significantly enhances the sorption properties of MgH_2_. A study on the catalytic mechanism revealed that in situ formations of metal oxides such as MgO, ZnO, and NiO during the heating process may help in improving the hydrogen storage performance of MgH_2_.

## 4. Conclusions

In this work, Ni_0.6_Zn_0.4_O samples were successfully synthesized via the solid-state method, and the catalytic effects of Ni_0.6_Zn_0.4_O on the hydrogen storage performance of MgH_2_ were systematically studied for the first time. Different weight percentages (5, 10, 15, and 20 wt.%) of Ni_0.6_Zn_0.4_O were milled together for 1 h, and the onset desorption temperature was reduced to a range of 280 °C to 305 °C, which is lower than pure MgH_2_ (418 °C) and milled MgH_2_ (341 °C). The absorption and desorption kinetics of MgH_2_ could be largely enhanced by the addition of 10 wt.% of Ni_0.6_Zn_0.4_O as an additive. The MgH_2_–10 wt.% Ni_0.6_Zn_0.4_O samples can absorb 6.50 wt.% of H_2_ in 1 h at 250 °C. Meanwhile, milled MgH_2_ can absorb only 4.10 wt.% of H_2_ under the same circumstances. For the desorption kinetics, the MgH_2_–10 wt.% Ni_0.6_Zn_0.4_O samples can release approximately 2.20 wt.% of H_2_ in 1 h at 300 °C, whereas pure MgH_2_ and milled MgH_2_ can only release releases <1.0 wt.% of H_2_ under the same conditions. From DSC and Kissinger desorption analyses, the apparent activation energy of the MgH_2_–10 wt.% Ni_0.6_Zn_0.4_O samples is 97 kJ/mol, resulting in a decrease of 36 kJ/mol compared with milled MgH_2_. Furthermore, the morphology becomes smaller and less agglomerated after the addition of 10 wt.% Ni_0.6_Zn_0.4_O. Smaller particles size provided more grain boundaries and larger surface area which benefited the diffusion path for hydrogen during the absorption and release process. From these results, it can be concluded that the reduction in particle size and the in situ generated (ZnO, NiO, and MgO) during the heating process played synergistic catalytic effects that boosted the hydrogen storage performance of MgH_2_.

## Figures and Tables

**Table 1:** Element of Ni_0.6_Zn_0.4_O samples.

Element	Mass (%)
Ni	61.88
Zn	17.08
O	21.04
Total	100.00

**Table 2:** Isothermal absorption kinetics curves from previous studies.

System	Temperature for Isothermal Absorption Kinetics (°C)	Absorption Capacity (wt.%)	Time (Min)	Refs.
MgH_2_ + 10 wt.% BaFe_12_O_19_	150	4.30	10	[46]
MgH_2_ + 10 wt.% MgFe_2_O_4_	200	5.50	10	[47]
MgH_2_ + 10 wt.% Co_2_NiO	320	2.50	1.7	[48]
MgH_2_ + Ni-50% Cu	300	5.24	30	[49]
MgH_2_–10 wt.% Ni_0.6_Zn_0.4_O	250	6.50	60	(this work)

**Table 3:** Onset desorption temperature, absorption capacity at 250 °C for 5 min and desorption capacity at 300 °C for 1 h.

	Onset Desorption Temperature (°C)	Absorption Capacity (wt.%)	Desorption Capacity (wt.%)
Pure MgH_2_	418	-	-
Milled MgH_2_	341	4.8	0.3
MgH_2_–5 wt.% Ni_0.6_Zn_0.4_O samples	280	4.1	2.7
MgH_2_–10 wt.% Ni_0.6_Zn_0.4_O samples	285	6.5	2.9
MgH_2_–15 wt.% Ni_0.6_Zn_0.4_O samples	305	6.5	4.3
MgH_2_–20 wt.% Ni_0.6_Zn_0.4_O samples	293	5.4	4.7

**Table 4:** Equation for kinetic models used for absorption and desorption kinetics of this study.

Integrated Equation	Model
A = kt	Surface-controlled (chemisorption)
[−ln(1 − α)]^1/2^ = kt	JMA, *n* = 2 (e.g., two-dimensional growth of existing nuclei with constant interface velocity)
[−ln(1 − α)]^1/3^ = kt	JMA, *n* = 3 (e.g., two-dimensional growth of existing nuclei with constant interface velocity)
1 − (1 − α)^1/3^ = kt	CV 2D: contracting volume, three-dimensional growth with constant interface velocity
1 − (2α/3) − (1 − α)^2/3^ = kt	CV 3D: contracting volume, three-dimensional growth diffusion controlled with decreasing interface velocity

Where t is time, k is a reaction rate constant and α is reacted fraction.

## Data Availability

The data presented in this study are available on request from the corresponding author.
